# Thrombotic microangiopathy triggered by podocytopathy 

**DOI:** 10.5414/CNCS110534

**Published:** 2021-10-04

**Authors:** Rita Veríssimo, Catarina Mateus, Ivo Laranjinha, Rita Theias Manso, Jorge Dickson, Margarida Gonçalves, Maria Augusta Gaspar, Domingos Machado

**Affiliations:** 1 Nephrology Department, Hospital de Santa Cruz, Centro Hospitalar Lisboa Ocidental, Lisbon,****and; 2 Pathology Department, Hospital Prof. Dr. Fernando da Fonseca, Lisbon,****Portugal

**Keywords:** thrombotic microangiopathy, nephrotic syndrome, minimal change disease

## Abstract

Thrombotic microangiopathy (TMA) is a rare group of diseases characterized by microangiopathic hemolytic anemia, thrombocytopenia, and target organ damage. It can be divided into primary and secondary TMA. Herein we report a case of TMA associated to a primary glomerular disease. We report the case of a 31-year-old Black male from Cape Verde admitted in March 2018 with nephrotic syndrome and upper gastrointestinal bleeding, the latter due to severe erythematous gastritis. He was discharged after clinical stabilization. The patient came to Portugal 8 months later. On admission, he presented with rapid deterioration of kidney function and hyperkalemia. The etiologic study revealed microangiopathic hemolytic anemia, nephrotic syndrome and microscopic hematuria. Immunologic study and viral serology were negative. ADAMTS13 activity and inhibitor testing were within normal range, genetic complement evaluation showed *CFH-H3* in homozygosity, functional complement studies revealed decreased function of alternative pathway. Kidney biopsy was consistent with the diagnosis of TMA, and electron microscopy was compatible with minimal change disease. Patient underwent plasmapheresis with resolution of hemolysis, fluid overload and recovery of renal function. Two months later, he presented with nephrotic syndrome and started prednisolone with remission. Six months later, the nephrotic syndrome relapsed, and it became steroid-, MMF-, and rituximab-resistant. Tacrolimus was initiated, achieving partial remission. Atypical hemolytic uremic syndrome is an uncommon disease and is rarely reported as secondary to glomerular diseases. This case showcases the challenges regarding treatment options in a resistant glomerulopathy and the implications of therapeutic choices and kidney outcomes with the coexisting TMA.

## Introduction 

Thrombotic microangiopathy (TMA) is a rare group of diseases characterized by microangiopathic hemolytic anemia, thrombocytopenia, and target organ damage [1, 2, 3, 4, 5]. TMA can be divided into primary (genetic and acquired) and secondary TMA [1, 2]. Primary genetic causes encompass: deficiency of ADAMTS13 (known as thrombotic thrombocytopenic purpura (TTP)); complement-mediated hemolytic uremic syndrome (HUS), also known as atypical hemolytic uremic syndrome (aHUS) [1, 2, 5]; cobalamin C hemolytic uremic syndrome, due to a rare autosomal recessive disorder of cobalamin metabolism [2, 5]; and mutations in *DGKE* (diacylglycerol epsilon), and in the *INF2* (inverted formin 2) gene [2]. Primary acquired TMA causes include: TTP secondary to autoantibodies to ADAMTS13 and complement-mediated HUS secondary to autoantibodies, most commonly against factor H (FH). Secondary TMA is a heterogeneous group that can be associated with various underlying conditions, such as infection, malignant hypertension, autoimmune disease, pregnancy, malignancy, drugs, inflammatory states, or rarely glomerular diseases [1, 2]. Genetic mutations in complement genes have been identified in many patients with secondary TMA, suggesting a possible overlap between primary and secondary TMA [1, 2, 7]. Herein we report a case of TMA associated to a glomerular disease. 

## Clinical case 

We report the case of a 31-year-old Black male from Cape Verde with no personal or family history of kidney disease and no use of prescribed or recreational drugs. In March 2018 the patient was diagnosed with nephrotic syndrome (edema, hypoalbuminemia, urinary protein-to-creatinine ratio (uPCR) of ~ 4,000 mg/g without hematuria or leukocyturia), and in May 2018 he was admitted to the hospital in Cape Verde, with worsening of the nephrotic syndrome and upper gastrointestinal bleeding. At the time, he had hemoglobin of 4.1 g/dL, normal platelet count, without signs of hemolysis (LDH, haptoglobin, and bilirubin were within the normal range). He had hyperlipidemia (total cholesterol 644 mg/dL, LDL 439 U/L, triglycerides 605 mg/dL), uPCR of ~ 13,000 mg/g without hematuria or leukocyturia, and normal potassium and kidney function. Endoscopic and histologic studies showed severe erythematous gastritis with no evidence of vasculitis, assumed as the underlying cause of the upper gastrointestinal bleeding. He was discharged after clinical stabilization, medicated with diuretics, proton-pump inhibitor, and ACE inhibitor. 

In November 2018, he was transferred to Portugal for further evaluation. At admission in a tertiary care hospital in Lisbon, he presented with rapid rise in serum creatine (sCr) (1.3 – 3.3 mg/dL in 4 months), fluid overload, and hyperkalemia (K 6.8 mmol/L). There was no evidence of infection, diarrhea, macroscopic hematuria, fever, or flank pain, and no prior history of oral or nasal ulcers, joint or pleuritic pain, skin rash, seizures, paresthesia, visual disturbances or other neurologic symptoms. The laboratory workout was compatible with microangiopathic hemolytic anemia (Hb 8.3 g/dL, platelet count 97×10^9^/L, haptoglobin < 10 mg/dL (30 – 200), LDH 448 U/L (135 225), and schistocytes in peripheral blood smear), nephrotic syndrome (urinary albumin-to-creatinine ratio (uACR) 5,193 mg/g, serum albumin 1.9 g/dL, triglycerides 474 mg/dL, cholesterol 555 mg/dL), and microscopic hematuria. The etiological study was unremarkable: ANCA, ANA, anti-GBM, and anti-dsDNA antibodies were negative; serum immunoglobulin, free light chain and complement (C3 and C4) levels were within normal range; viral serologies (hepatitis B, C, and HIV) were negative; direct Coombs test was negative. 

**Figure 1:**
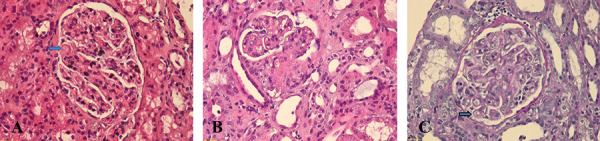
A: (H & E × 20) Membranoproliferative glomerulonephritis (MPGN)-like pattern with hyperlobulated glomerular tufts, light proliferation of mesangial and endothelial cells, and double contouring of basement membranes (arrow). B: (H & E × 20) MPGN-like pattern, presence of an endoluminal thrombus in the vascular pole. C: (PAS × 20) MPGN-like pattern, presence of double contour of basement membrane (arrow).

A kidney biopsy was performed, which showed: 21 glomeruli, with membranoproliferative glomerulonephritis (MPGN)-like pattern with hyperlobulated glomerular tufts, light proliferation of mesangial and endothelial cells, and double contouring of basement membranes, without evidence of glomerulosclerosis or crescentic glomerulonephritis; glomerular capillary congestion with intraluminal thrombus; no onion skin-like changes were observed in the blood vessels in this biopsy; mild tubular atrophy and interstitial fibrosis; immunofluorescence of sectioned frozen tissue was negative (IgG, IgA, IgM, C3, C1q, fibrinogen, albumin, κ and λ-light chains) (Figure 1). 

In view of the TMA diagnosis, plasma exchange (PE) was started, using a PrismaFlex monitor with TPE 2000 filter (Gambro/Baxter Int., Lund, Sweden) with a central venous catheter as vascular access and unfractionated heparin (UFH) as anticoagulant. The plasma volume to treat was calculated using the Kaplan formula (4.47 L of plasma). The patient completed 12 sessions of PE in 18 days, with fresh frozen plasma as the replacement fluid. The treatment resulted in resolution of hemolysis, improvement of platelet count and kidney function, but persistence of nephrotic proteinuria (Table 1). The patient was discharged on December 2018 with no significant edema. 

ADAMTS13 activity and inhibitor testing were within normal range; classic complement pathway function (CH50) was normal, but alternative complement pathway function was decreased (AH50 43%, reference range >70%); complement factors and regulators were within normal range; genetic analysis showed pathologic variants in *ADAMTS13* gene in heterozygosity (exon 16 c.1874G>A p. Arg625HIS and exon 25 c.3541G>A p. Gly1181Arg) and *CFH-H3* in homozygosity (risk haplotype). 

Three weeks after discharge, the patient presented with worsening of the nephrotic syndrome. 

**Figure 2:**
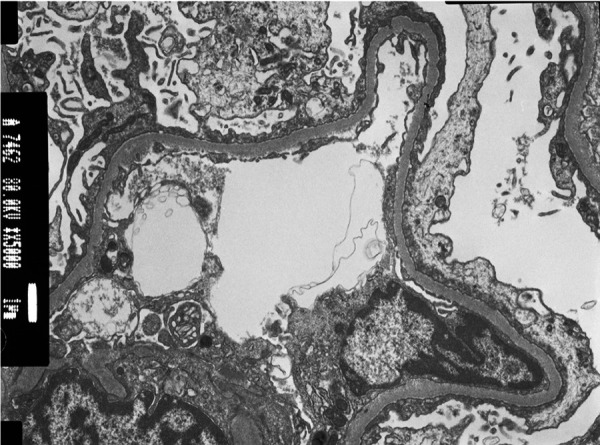
Electron microscopy × 5,000: Extensive podocyte foot process effacement in a preserved basement membrane area, compatible with podocytopathy.

At readmission, electron microscopy results from the previous biopsy were available and showed some capillary wall with basement “neomembran” involving some fibrin deposits and some detritus and fluffy material and diffuse foot process effacement, compatible with minimal change disease (MCD) (Figure 2). Prednisolone 1.5 mg/kg every other day was started, with partial remission of the nephrotic syndrome within 2 weeks (serum albumin 3.5 g/dL, uACR 300 mg/g, and no fluid overload). 

A month after the beginning of treatment, the patient stopped taking the medication of his own accord and, when re-evaluated, he presented with recurrence of the nephrotic syndrome (uACR 3,800 mg/g, decrease of serum albumin from 3.5 to 2.2 mg/d, and fluid overload). Prednisolone was restarted in the previous dose, but, despite adequate compliance, there was no response to glucocorticoids. A kidney biopsy was repeated in June 2019, 7 months after the diagnosis, which showed 20 glomeruli, 3 of them sclerosed and the rest without histological alterations; mild tubular atrophy and interstitial fibrosis; immunofluorescence of sectioned frozen tissue negative (IgG, IgA, IgM, C3, C1q, fibrinogen, albumin, Kappa and Lambda light chains). Electron microscopy was not performed, and a diagnosis of MCD was assumed. A genetic analysis was performed to search for a genetic mutation that could lead to podocytopathy, and it only showed a variant in *FAT1* gene in heterozygosity (c. 11771G>T, p.(Arg3924Leu)) which is not associated with pathogenicity in most studies/reports. 

A steroid-resistant MCD was assumed, and, due to the risk of relapse of TMA with calcineurin inhibitors, a second-line therapy with mycophenolate mofetil (MMF) was started, with progressive increase in dosage up to 1.5 g twice daily. Four months later, there was no response to MMF therapy. At this time, MMF was discontinued and rituximab was administered (1 g at day 0 and day 14). Despite confirmation of CD19-lymphocyte depletion, no clinical improvement was observed (weight gain despite optimization of diuretic therapy, serum albumin 1.3 g/dL, worsening dyslipidemia, and uPCR 8,579 mg/g). After careful consideration, tacrolimus was started, with target trough levels of 4 – 6 ng/mL and close monitoring of early signs of hemolysis. 

## Follow-up and outcome 

During the entire follow-up there were no signs of TMA recurrence. Four months after the beginning of calcineurin inhibitor therapy, the patient has no edema, serum albumin is near normal range and uACR dropped to 2,450 mg/g. 

## Discussion 

aHUS is responsible for 5 – 10% of cases in children and for the majority of adult cases, with an annual incidence of 2 cases/million people [1, 5]. In ~ 60% of cases, it is linked to mutations in complement genes encoding proteins involved in the complement alternative pathway. Some patients have genetic risk haplotypes such as CFH-H3 (which identifies the CFHtgtgt risk haplotype) or MCPaaggt and may not have clinically apparent HUS until a condition (such as malignancy, drugs, transplantation, systemic diseases, and infection) triggers an acute TMA episode [8, 9, 10]. 

aHUS being precipitated by glomerular diseases is uncommon. In 2013, a case series and review of literature was published, which identified several cases of aHUS triggered by different glomerular diseases. These included 36 vasculitis syndromes; 20 MPGN; 10 focal and segmental glomerulosclerosis (FSGS); 7 membranous glomerulonephritis (MGN), and 1 MCD [10]. An association with IgA nephropathy (IgAN) has also been described in case reports and in two retrospective studies, which demonstrated that ~ 20 – 25% of IgAN biopsies have histological microangiopathic lesions, although most patients also had uncontrolled hypertension [2, 11, 12, 13, 14, 15, 16], and most of these were histologic lesions without biochemical or clinical manifestations of TMA [17]. 

**Figure 3:**
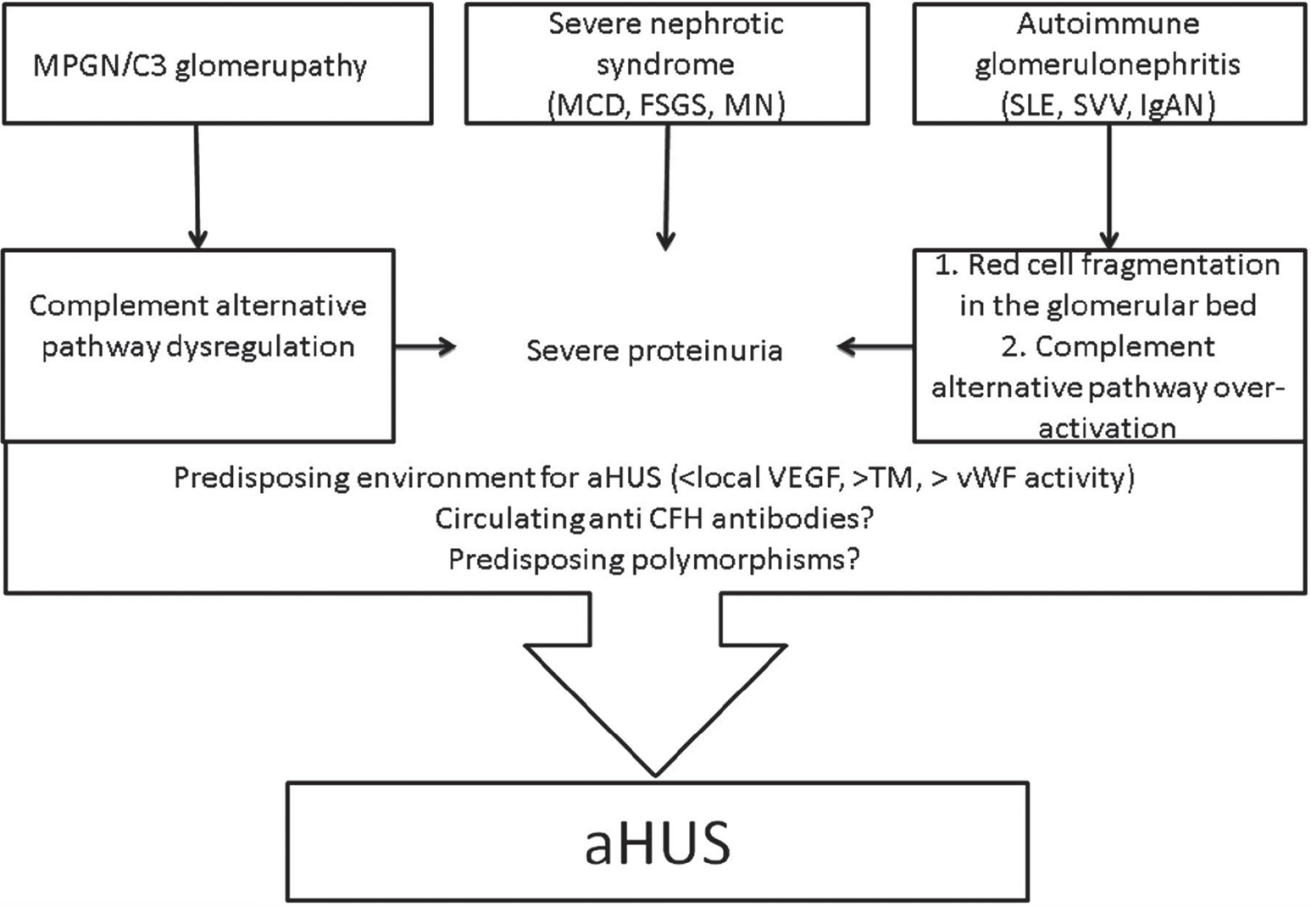
Proposed mechanism of glomerulopathies-associated atypical hemolytic uremic syndrome. Reprinted from “Atypical haemolytic uraemic syndrome with underlying glomerulopathies. A case series and a review of the literature.” by Manenti L et al., Nephrol Dial Transplant. 2013; 28: 2246-2259. Copyright © 2013, Oxford University Press. Reprinted with permission.

A bidirectional relationship between glomerular diseases and TMA has been suggested. It has been proposed that the abnormal complement activation can lead to podocyte damage and dysfunction. The podocyte injury can, in turn, trigger the development of TMA [3] in consequence of the imbalance of coagulation (by the loss of anticoagulants through the defective glomerular filtration barrier and procoagulant factors synthesis by the liver). Nephrotic syndrome can cause a plasma soluble thrombomodulin, and von Willebrand factor levels that can contribute to the development of TMA (Figure 3) [3, 9, 17, 18, 19]. 

With regard to the case presented above, the patient had been diagnosed with nephrotic syndrome a few months before the TMA was clinically apparent. The authors’ premise is that in this TMA-predisposed patient (with *CFH-H3* in homozygosity), the TMA developed after the second hit, which was the nephrotic syndrome. The hypothesis of TTP was discarded despite the presence of the polymorphisms in *ADAMTS13* gene, because its activity was within the normal range. It has been suggested that those mutations (*CFH-H3* and *ADAMTS13* genes) could have a synergistic effect that increases the risk of developing aHUS [20]. 

Concerning the biopsy results, the first describes a MPGN-like pattern that was not present in the second biopsy, after resolution of the TMA. Currently, MPGN is classified as: (i) immune-complex mediated, which can be seen in hepatitis C and other infections, autoimmune disorders or monoclonal gammopathy; (ii) complement mediated, which can result in dense-deposit disease or C3 glomerulonephritis (C3GN); (iii) MPGN without immune complexes or complement activation [21, 22, 23]. Our patient had a genetic variation in complement factor H gene, which could be related to a dysregulation of the alternative pathway, and it could be hypothesized that he could have an immune-complex-mediated MPGN, although the negative immunofluorescence and the absence of this pattern in the subsequent biopsy are against this option. MPGN without immune complexes or complement activation usually occurs due to endothelial injury and can be seen in the healing or chronic phase of TMA. In this type of MPGN, immunoglobulin and complement are not present in immunofluorescence, and electron-dense deposits are absent in the mesangium or capillary walls on electron microscopy [21, 23]. This can explain the presence of this MPGN-like pattern, absent in the second biopsy, which only revealed a podocytopathy. Uncontrolled hypertension, antiphospholipid syndrome, or any other cause of this type of MPGN were excluded. 

Approximately 10% of MCD patients have a steroid-resistant disease. After confirming compliance and absence of over-the-counter medications, some authors suggest repeating kidney biopsy to exclude FSGS diagnosis. Although it will not change the treatment choice, it gives important information about the long-term prognosis. Genetic forms of MCD are rare and should be investigated in these patients. Regarding treatment, it should follow the same guidelines used for FSGS [24, 25]. The first treatment option in this type of disease is calcineurin inhibitors (CNI), with cyclosporine being more studied than tacrolimus [25]. In our case, initially we decided against this option due to the risk of TMA relapse with these agents. This association is stronger with cyclosporine use, with an incidence of 2 – 5%, while tacrolimus-induced TMA has an incidence of ~ 1% [26]. Cyclophosphamide (CYC) has been studied in FSGS glucocorticoid resistant disease, with half of the patients achieving remission, but its important toxicity should be weighed against the beneficial effects, particularly in younger patients [27, 28, 29]. MMF and azathioprine therapy has been described in some case reports and small uncontrolled studies, but with lack of supporting evidence, and rituximab use has been reported in small studies, with inconsistent results [27, 30, 31, 32, 33, 34, 35, 36, 37]. 

In our case, since the patient had a real possibility of TMA relapse with CNI, and CYC has important associated toxicity, we decided to start MMF. Faced with the unresponsiveness of the patient to this second-line treatment, and in light of the positive, although small, studies regarding the use of rituximab, we decided to use it as a third therapeutic option. Finally, when the patient was found steroid-, MMF-, and rituximab-resistant, there was limited choice remaining, and tacrolimus was initiated due to the lower risk of TMA relapse, achieving partial remission. The absence of complete remission and the patient’s African ancestry highlights the probability of a possible misdiagnosis – in this case, a missed FSGS by sampling error in the kidney biopsies performed despite the presence of adequate glomeruli number in both biopsies. 

## Conclusion 

aHUS is an uncommon disease and is rarely reported in patients with glomerular diseases. As the patient first presented with nephrotic syndrome, which is associated with the development of HUS in predisposed patients, our hypothesis is that the patient’s podocytopathy led to TMA. In our patient, this genetic predisposition is the presence of *CFH-H3*, a risk haplotype, in homozygosity, which can predispose to HUS. 

This case also showcases the challenges regarding the treatment options in resistant glomerulopathy. The concurrent diagnosis of TMA limits our therapeutic choices and has important implications in kidney outcomes. 

## Acknowledgment 

We thank L. Manenti et al. for the permission to use Figure 2 of their article. 

## Funding 

None to declare. 

## Conflict of interest 

None declared. 

**Table 1.** Evolution of laboratorial parameters with plasma exchange (PE) treatments.

Day since the beginning of PE	Hemoglobin (g/dL)	Serum creatinine (mg/dL)	Serum albumin (g/dL)	Urinary albumin-to- creatinine ratio (mg/g)
1	8.7	3	1.5	5,193
3	7.1	2.93	2.2	–
6	8.4	1.52	2.2	–
9	7.5	1.41	2.4	–
12	8.1	1.21	2.3	–
16	8.9	0.93	2.8	–
18	10.6	0.89	3.1	7,458
